# Determinants of Institutional Delivery among Childbearing Age Women in Western Ethiopia, 2013: Unmatched Case Control Study

**DOI:** 10.1371/journal.pone.0097194

**Published:** 2014-05-08

**Authors:** Tesfaye Regassa Feyissa, Gebi Agero Genemo

**Affiliations:** 1 College of medical and health sciences, Wollega University, Nekemte, Oromia, Ethiopia; 2 School of Health Sciences, Adama University, Asella, Oromia, Ethiopia; Iran University of Medical Sciences, Iran (Republic of Islamic)

## Abstract

**Background:**

Place of delivery is a crucial factor which affects the health and wellbeing of the mother and newborn. Institutional delivery helps the women to access skilled assistance, drugs, equipment, and referral transport. Even though 34% of pregnant women received at least one antenatal care from a skilled provider in Ethiopia by 2013, institutional delivery was 10%. The main objective of the study was to assess determinants of institutional delivery in Western Ethiopia.

**Methods:**

Retrospective unmatched case control study design was used to assess determinants of institutional delivery in Western Ethiopia from September to October 2013. A total of 320 respondents from six districts of East Wollega zone, West Ethiopia were included. Data were collected using pretested and structured questionnaires. Data were entered and cleaned by Epi-info then exported and analyzed using SPSS software. Statistical significance was determined through a 95% confidence level.

**Results:**

Education [Adjusted Odds Ratio (AOR) (95% Confidence Interval (CI)) = 2.754(1.510–8.911)], family size [AOR (95% CI) = .454(.209–.984)], residence [AOR (95% CI) = 3.822 (1.766–8.272)] were important predictors of place of delivery. Four or more antenatal care [(ANC) (AOR (95% CI) = 2.914(1.105–7.682)], birth order [(AOR (95% CI) = .136(.054–.344), age at last delivery [(AOR (95% CI) = 9.995(2.101–47.556)], birth preparedness [AOR (95% CI) = 6.957(2.422–19.987)], duration of labour [AOR (95% CI) = 3.541(1.732–7.239)] were significantly associated with institutional delivery. Moreover service related factors such as distance from health institutions [AOR (95% CI) = .665(.173–.954)], respondents’ awareness of skill of health care professionals [AOR (95% CI) = 2.454 (1.663–6.255)], mode of transportations [AOR (95% CI) = .258(.122–.549)] were significantly associated with institutional delivery.

**Conclusions and Recommendations:**

Policy makers, health service organizations, community leaders and other concerned bodies have to consider the predictors of institutional delivery like education, birth order, antenatal care utilization and residence to improve institutional delivery in the area.

## Introduction

The fifth Millennium Development Goal (MDG) was to reduce maternal mortality ratio (MMR) by 3/4^th^ between 1990 and 2015. The maternal mortality ratio of Ethiopia was 676 deaths per 100,000 live births by 2011 compared to 673 deaths per 100,000 live births by 2005. Skilled birth attendance is correlated with lower MMR. At least 15% of all births in the population should take place in basic or comprehensive emergency obstetrics facilities. However, the proportion of births with a skilled attendant is 10% in Ethiopia. Therefore identifying the determinants of skilled attendance for delivery is a priority area to give recommendations [1], [2], [3], [4].

Delivery by skilled birth attendance serves as an indicator of progress towards reducing maternal mortality [5]. Place of delivery is a crucial factor which affects the health and wellbeing of the mother and the newborn. It’s usually a joyful event when women give birth to a baby she wants. However, birth is a critical time for the health of the mother and baby. If problems may arise during labour and delivery not treated properly and effectively can lead to ill health and even death of one or both of them [2], [3].

Childbirth takes place in different forms throughout the world depending on the cultural contexts of each community. In some areas mothers go through the process of unattended child birth. A few seek from a midwives and obstetrician, but two thirds of birth in the world are assisted by traditional birth attendants who are not trained [2].

Trends documenting the change in the proportion of births accompanied by a skilled attendant over the last 15–20 years offer no indication that adequate change is imminent. To reduce maternal mortality rapidly in regions where births in the home without skilled birth attendants are common, governments and community-based organizations could implement a cost-effective strategies [4].

Childbirth is a risk producing event. Timely and adequate medical care for women who experience obstetric complication is an option for mitigating the risk. Women are encouraged to deliver their babies in health facilities as a strategy to implement maternal health outcomes [6], [7], [8], [9], [10].

Birth is profoundly affected by the environment in which it takes place. The home and birth center environments have distinctive features that support healthy women experiencing normal birth. The rural and urban women who could not afford medical insurance or were not able to pay cash were attended by traditional birth attendants [9], [10].

Utilization of health service is affected by multitudes of factors including not only availability but distance, cost and quality of service, but also by socioeconomic factors and personal health beliefs [7], [11], [12].

There were systematic differences in place of delivery and type of attendance at delivery by age of the mother and the order of the birth and also by background and standard of living of the woman. But socio-economic factors have been shown to be of greater importance in determining health service use than demographic factors [2], [13].

Significant reductions in maternal mortality could be achieved if institutional delivery is available to, and used by all women. To accomplish such a goal, targeting factors which affect institutional delivery is essential. However, determinants of institutional delivery were not well understood in Western Ethiopia. Thus, the objective of study was to assess determinants of institutional delivery in Western Ethiopia.

## Materials and Methods

Retrospective unmatched case control study was conducted in East Wollega zone, Oromia regional state, western Ethiopia from September to October 2013. East Wollega zone is one of the 17 zones of Oromia regional state with total population of 1,230,402; 615,641 are females. Eighty six percent of the population leave in rural areas (1,061,120) [14].

The source population for the study was all women who gave birth in last five years in East Wollega Zone, while the study population was women who were selected by consecutive sampling technique from six districts in East Wollega Zone.

Cases were women who gave birth to their last child in health institutions in the last five years. Controls were women who give birth to their last child at home in the last five years in East Wollega zone.

Inclusion criteria for both cases and controls were women aged 15–49 years who gave birth in the last five years and fulfills the definition of cases and controls. Women with mental illness and severe illness were excluded from the study because it was considered that they could not give necessary information.

The sample size was calculated using EPI info software 3.5.1 version using following assumptions; 40% proportion of women were living in rural areas among women who give a birth at health institutions (cases) and 2.25 odds ratio to detect case (OR) [15], with 80% power and 5% type I error and ratio of cases to controls was 1∶3. The calculated sample size was 320 (80 cases and 240 controls). Those women fulfilling inclusion criteria were recruited until sample size was achieved. For each case three consecutive controls were selected from the community.

The questionnaire was initially prepared in English, translated to local language Afan Oromo, and back to English by different individuals to check its consistency. It was then pre-tested on 5% of the sample. The questionnaire was then assessed for its clarity, completeness. Some skip patterns were then corrected; questions difficult to ask were rephrased. The questionnaire had three parts. The first part was socio-demographic factors that encompass age, occupation, educational status, monthly family income, family size, residence, and communication materials. The second part was reproductive factors like ANC utilization, pregnancy related complications, and obstetrics history. The third part was programmatic factors includes infrastructure, transportations.

Six bachelor holder Nurse/midwife data collectors and three master’s holder supervisors were recruited. Two days training was given focusing on the objective of the study and value of collecting the actual data. The structured questionnaire was discussed in detail going through every question and clarification was provided.

A field manual was prepared for the supervisors and data collectors for use during data collection. All filled questionnaires were checked daily for completeness, accuracy, clarity and consistency by the supervisors and the principal investigators and necessary corrections and changes were made. Completeness and consistency of variables during data entry and analysis was checked using frequency distributions, cross tabulations, sorting in ascending and descending order.

Data were entered and cleaned using Epi Info software, and then exported to statistical package for social science (SPSS) software for analysis. Bivariate analysis between dependent and independent variables was performed separately using binary logistic regression. The strength of association between dependent variable and independent variables (covariates) was expressed in odds ratio (OR) through 95% confidence interval. Variables which have association in bivariate analysis were included in multivariate analysis. Finally multivariate analysis using forward stepwise multiple logistic regression technique was done to evaluate independent effect of each variable on institutional delivery by controlling the effect of others. Statistical significance was determined through a 95% confidence interval.

Ethical approval was obtained from Ethical Board Committee, Wollega University. The purpose of the study was clearly explained, informed oral consent was maintained and confidentiality was ensured. Oral consent was obtained because majority of study subjects were illiterate. Data collectors read the consent for respondents and they mark if the respondents agree. The consent form and information sheet was approved by the ethical board committee. A formal letter for permission and support was written to the respective administrator office. The purpose of the study was clearly explained to concerned bodies. The purpose and process of the study was explained to all participants. Before informed consent was obtained, the respondents were told that they have the right to be involved or not to be involved in the study.

## Results

### Socio-demographic Characteristics of the Study Participants

A total of 320 (80 cases and 240 controls) respondents were interviewed. Mean age for cases and controls was 24.5 and 25 years respectively. Regarding education, 27 (33.8%) of cases and 168 (70.0%) of controls were not attended formal education. Cases and controls who were farmers were 27 (33.8%) and 157 (65.4%) respectively. Median family income for cases and controls was 1000 Ethiopian birr (about 53 US $) and 750 Ethiopian birr (about 40 US $) respectively. As shown in table 1 there was significant difference between cases and controls in their age, educational status, occupation, wealth quintile, residence, family size (Table 1).

**Table 1:** Socio-demographic characteristics of study participants in Western Ethiopia, October 2013.

Characteristics		Cases (%)	Controls (%)	chi square, df, p value
**Age**	15–19	6 (7.5)	13 (5.4)	χ^2^ = 11.097
	20–24	34 (42.5)	68 (28.3)	df = 4
	25–29	28 (35.0)	83 (34.6)	p-value = .025
	30–34	5 (6.2)	45 (18.8)	
	35–49	7 (8.8)	31 (12.9)	
**Mean age (in years) (±SD)**		24.5 (**±**5.6)	25 (**±**5.6)	
**Educational status**	illiterate/informaleducation	27 (33.8)	168 (70.0)	χ^2^ = 53.051
	primary (1–8)	30 (37.5)	63 (26.2)	df = 2
	Secondary and above	23 (28.8)	9 (3.8)	p-value = .000
**Ethnicity**	Oromo	72 (90.0)	215 (89.6)	χ^2^ = .011
	Others*	8 (10.0)	25 (10.4)	df = 1, p-value = .915
**Occupation**	Farmer	27 (33.8)	157 (65.4)	χ^2^ = 42.985
	Housewife	24 (30.0)	47 (19.6)	df = 3
	Employed	18 (22.5)	7 (2.9)	p-value = .000
	Others**	11 (13.8)	29 (12.1)	
**Wealth quintile**	Lowest	12 (15.0)	49 (20.4)	χ^2^ = 14.625
	Second	11 (13.8)	54 (22.5)	df = 4
	Middle	13 (16.2)	51 (21.2)	p-value = .006
	Fourth	14 (17.5)	44 (18.3)	
	Highest	30 (37.5)	42 (17.5)	
**Median family income (ETB)**		1000 ETB	750 ETB	
**Residence**	Urban	54 (67.5)	57 (23.8)	χ^2^ = 50.692, df = 1, p-value = .000
	Rural	26 (32.5)	183 (76.2)	
**Family size**	1–3	33 (41.2)	39 (16.2)	χ^2^ = 23.781
	4–5	32 (40.0)	110 (45.8)	df = 2
	6 and above	15 (18.8)	91 (37.9)	p-value = .000
**Health workers** **visited you in** **the last 12 months**	Yes	50 (62.5%)	141 (58.8%)	χ^2^ = .351
	No	30 (37.5%)	99 (41.2%)	df = 1p-value = .554

*Amhara, Gurage, Tigire.

**House maid, private self-employment, retirement, merchant, daily laborer.

### Socio-demographic Determinants of Institutional Delivery

On bivariate analysis, age of the mother, family size, occupational status, educational status, residence, monthly family income, possessing radio/television were the factors found to be significantly associated with institutional delivery service utilization.

In a multivariate model, secondary and above education (AOR: 2.754, 95% CI: 1.510–8.911), family size of 4–5 (AOR: .454, 95% CI: .209–.984), living in urban areas (AOR: 3.822, 95% CI: 1.766–8.272) were the predictors of institutional delivery (Table 2).

**Table 2:** Socio-demographic determinants of institutional delivery in Western Ethiopia, October 2013.

Variables	Categories	Cases (institutionaldelivery) (%)	Controls (home delivery) (%)	CrudeOR (95% CI)	AdjustedOR (95% CI)
Age	15–19	6 (7.5)	13 (5.4)	1	1
	20–24	34 (42.5)	68 (28.3)	1.083 (.379–3.100)	1.089 (.317–3.744)
	25–29	28 (35.0)	83 (34.6)	.731 (.254–2.105)	1.030 (.277–3.827)
	30–34	5 (6.2)	45 (18.8)	**.241 (.063–.917)**	.382 (.077–1.895)
	35–49	7 (8.8)	31 (12.9)	.489 (.138–1.739)	1.034 (.219–4.890)
Education level	Illiterate/ableto read and write	27 (33.8)	168 (70.0)	1	1
	Primary (1–8)	30 (37.5)	63 (26.2)	**2.963 (1.634–5.373)**	1.433 (.712–2.881)
	Secondaryand above	23 (28.8)	9 (3.8)	**15.901 (6.654–38.0)**	**2.754 (1.510–8.911)**
Occupation	Farmer	27 (33.8)	157 (65.4)	1	1
	Housewife	24 (30.0)	47 (19.6)	**2.969 (1.567–5.626)**	.861 (.356–2.082)
	Employed	18 (22.5)	7 (2.9)	**14.952 (5.703–39.200)**	2.015 (.541–7.505)
	Others*	11 (13.8)	29 (12.1)	2.206 (.986–4.935)	.727 (.267–1.980)
Family size	1–3	33 (41.2)	39 (16.2)	1	1
	4–5	32 (40.0)	110 (45.8)	**.344 (.187–.632)**	**.454 (.209–.984)**
	6 and above	15 (18.8)	91 (37.9)	**.195 (.095–.399)**	.446 (.176–1.126)
Monthlyfamily income	<600 birr	23 (28.8)	103 (42.9)	1	1
	600–1499	27 (33.8)	95 (39.6)	1.273 (.683–2.371)	1.106 (.537–2.278)
	1500 and above	30 (37.5)	42 (17.5)	**3.199 (1.668–6.134)**	1.344 (.576–3.136)
Residence	Urban	54 (67.5)	57 (23.8)	**6.668 (3.831–11.607)**	**3.822 (1.766–8.272)**
	Rural	26 (32.5)	183 (76.2)	1	1
PossessingRadio and TV	Yes	64 (80.0)	135 (56.2)	**3.111 (1.700–5.693)**	1.677 (.816–3.449)
	No	16 (20.0)	105 (43.8)	1	1

*Merchant, daily laborer.

### Antenatal Care and Reproduction Related Determinants of Institutional Delivery

On bivariate analysis; number of ANC visits, birth order, pregnancy related problems, duration of labour, and birth preparedness were the factors found to be significantly associated with institutional delivery service utilization.

In a multivariate model, four or more ANC (AOR: 2.914, 95% CI: 1.105–7.682), 2–3 birth order (AOR: .214, 95% CI: .105–.436), greater than 3 birth order (AOR: .136, 95% CI: .054–.344), 6–11 hours duration of labour (AOR: 3.197, 95% CI: 1.444–7.080), 12 hrs and above duration of labour (AOR: 3.541, 95% CI: 1.732–7.239), age at last delivery 35–49 (AOR: 9.995,95% CI: 2.101–47.556) and birth preparedness (AOR: 6.957,95% CI: 2.422–19.987) were important predictors institutional delivery (Table 3).

**Table 3:** Antenatal care and reproduction related determinants of institutional delivery in Western Ethiopia, October 2013.

Variables	Categories	Cases(institutionaldelivery) (%)	Controls(homedelivery) (%)	CrudeOR (95% CI)	AdjustedOR (95% CI)
Number of ANCreceived	No ANC received	7 (8.8)	72 (30.0)	1	1
	1–3	25 (31.2)	89 (37.1)	**2.889 (1.182–7.063)**	1.628 (.598–4.433)
	4 and above	48 (60.0)	79 (32.9)	**6.250 (2.658–14.693)**	**2.914 (1.105–7.682)**
Birth order	1	45 (56.2)	58 (24.2)	1	1
	2–3	24 (30.0)	106 (44.2)	**.292 (.162–.526)**	**.214 (.105–.436)**
	4 and more	11 (13.8)	76 (31.7)	**.187 (.089–.392)**	**.136 (.054–.344)**
Age atlast delivery	15–19	17 (21.2)	38 (15.8)	1	1
	20–34	57 (71.2)	190 (79.2)	.671 (.352–1.277)	1.643 (.729–3.705)
	35–49	6 (7.5)	12 (5.0)	1.118 (.359–3.476)	**9.995 (2.101–47.556)**
Pregnancyrelated Problems	Yes	21 (26.2)	32 (13.3)	**2.314 (1.242–4.308)**	**2.209 (1.028–4.744)**
	No	59 (73.8)	208 (86.7)	1	1
Labour duration	less than 6 hrs	30 (37.5)	138 (57.5)	1	1
	6–11 hrs	19 (23.8)	45 (18.8)	1.942 (.998–3.780)	**3.197 (1.444–7.080)**
	12 hrs and above	31 (38.8)	57 (23.8)	**2.502 (1.388–4.510)**	**3.541 (1.732–7.239)**
Birthpreparedness	Yes	75 (93.8)	152 (63.3)	**8.5299 (3.323–21.895)**	**6.957 (2.422–19.987)**
	No	5 (6.2)	87 (36.6)	1	1

### Programmatic Related Determinants of Institutional Delivery

On bivariate analysis, distance from the nearby health institution, number of health care providers, time to reach health institution, average waiting time at health institution and mode of travel to the nearest health institutions were the factors found to be significantly associated with institutional delivery service utilization.

In a multivariate model, distance ≥10 km (AOR: .665, 95% CI: .173–.954), skill of health care professionals (AOR: 2.454, 95% CI: 1.663–6.255), transportation on foot (AOR: .258, 95% CI: .122–.549) were significantly associated with institutional delivery (Table 4).

**Table 4:** Programmatic related determinant factors of institutional delivery in Western Ethiopia, October 2013.

Variables	Categories	Cases (institutional delivery)(%)	Controls(home delivery)(%)	CrudeOR (95% CI)	AdjustedOR (95% CI)
Distance fromhealth institution	less than 5 KM	46 (57.5)	97 (40.4)	1	1
	5-<10 KM	7 (8.8)	48 (20.0)	**.308 (.129**–**.732)**	.440 (.177–1.096)
	10 Km and above	10 (12.5)	76 (31.7)	**.277 (.131**–**.586)**	**.665 (.173**–**.954)**
	Don’t Know	17 (21.2)	19 (7.9)	1.887 (.898–3.964)	**3.719 (1.480–9.346)**
Number of healthcare providers	Don’t know	20 (25.0)	80 (33.3)	1	1
	1–2	41 (51.2)	133 (55.4)	1.233 (.675–2.252)	1.333 (.670–2.655)
	3 and above	19 (23.8)	27 (11.1)	**2.815 (1.310–6.046)**	1.826 (.739–4.511)
Skill of healthcare providers	High	52 (65.0)	116 (48.3)	**2.421 (1.144–5.124)**	**2.454 (1.663–6.255)**
	Medium	18 (22.5)	70 (29.2)	1.389 (.593–3.251)	1.681 (.604–4.679)
	Poor	10 (12.5)	54 (22.5)	1	1
Time to reachhealth institution	<1 hour	68 (85.0)	154 (64.2)	**3.864 (1.321–11.299)**	3.554 (.884–14.283)
	1-<2 hour	8 (10.0)	51 (21.2)	1.373 (.384–4.912)	2.054 (.461–9.145)
	>2 hour	4 (5.0)	35 (14.6)	1	1
Average waitingtime at healthinstitution	1–15 minutes	43 (53.8)	85 (35.4)	1	1
	16–30 minutes	18 (22.5)	73 (30.4)	**.487 (.259**–**.918)**	.561 (.277–1.137)
	31–60 minutes	10 (12.5)	48 (20.0)	**.412 (.190**–**.893)**	.541 (.227–1.291)
	>60 minutes	9 (11.2)	34 (14.2)	.523 (.230–1.189)	1.012 (.370–2.768)
Mode of travelto thenearest health institutions	Foot	58 (72.5)	218 (90.8)	**.266 (.138**–**.514)**	**.258 (.122–.549)**
	Others*	22 (27.5)	22 (9.2)	1	1

*car, horse, mule, cart.

## Discussions

The proportion of women who delivered with the assistance of a skilled birth attendant is one of the indicators in meeting the fifth MDG. In almost all countries where health professionals attend more than 80% of deliveries, MMR is below 200 per 100,000 live births [16]. In Ethiopia, skilled health professionals attend very few births. Considering the facts, this study examined factors determining institutional delivery service utilization in Western Ethiopia.

To ensure skilled attendance at birth, the international community set a target of 80% by 2005, 85% by 2010 and 90% coverage by 2015. However, in 2008 only 65.7% of all women were attended by a skilled attendant during pregnancy, childbirth and immediately postpartum globally with some countries having less than 20% coverage. However, skilled attendance in Ethiopia was 10% by 2011 [15], [17], [18], [19], [20].

In this study educational status of mothers was one of the important predictor in determining institutional delivery in multivariate analysis. Women who attended secondary and above education were about three times (AOR: 2.754, 95% CI: 1.510–8.911) more likely to give birth at health institution. The data is consistent with studies from developing countries [21], [22], [23]. It is also consistent with studies from different regions of Ethiopia [15], [24], [25], [26], [27], [28]. Education is a key factor in improving institutional delivery, but it is challenge for countries like Ethiopia where more than half of the women (51%) had no formal education [1]. Education is likely to enhance female autonomy so that mothers develop greater confidence and capabilities to make decisions regarding their own health. Educated women are more likely to be aware of difficulties during pregnancy and as a result, they are more likely to use institutional health services.

Mothers living in urban areas (AOR: 3.822, 95% CI: 1.766–8.272) were more likely to give birth in health institution. Ethiopia demographic and health survey report (2011)[1] showed that living in an urban area helps to seek modern health care. The reason may be due to health facilities accessibility in urban areas than rural areas.

If health facilities are not in walking distance, rural mothers are less likely to afford transportation cost. In many instances even if they can afford to pay the transportation fee, the vehicle may not be available at the time they need it. This indicates the difference in access especially in terms of physical distance. Findings might be due the fact that in urban areas the proportion of mothers with education is higher, accessibility of the services with minimal distance and transport, and mothers could have better decision making autonomy, good knowledge of pregnancy and delivery complications and better access to information than rural mothers.

The finding of the study showed that birth preparedness had association with institutional delivery (AOR: 6.957, 95% CI: 2.422–19.987). Study from South-Western Uganda indicated that birth preparedness was also associated with being assisted by skilled birth attendant at the time of delivery [23]. The practice of birth preparedness should be encouraged and expanded during the antenatal utilization and other ongoing conversation with community.

The study highlight that higher the birth orders the less likely the women were to give birth at health institution. Studies from low income countries [21], [23] suggest that birth order was consistently associated with facility based delivery. It is also consistent with studies from Ethiopia [26], [29]. The likely reason is that women with more children perceive delivery as a normal process and develop the confidence to give birth at home. It implies that family planning has a significant role in improving institutional delivery and reducing maternal death.

Another predictor of institutional delivery is reported pregnancy related complications (AOR: 2.209, 95% CI: 1.028–4.744**)**. In the Philippines, health problems identified at antenatal care were found to be statistically associated with facility-based delivery [21]. In Dodota district, Oromia region, pregnancy related health problems showed significant positive association with utilization of institutional delivery services [28]. It implies that health care utilization is often after developing some health problems.

Duration of labour (AOR: 3.541, 95% CI: 1.732–7.239) were significantly associated with institutional delivery. Studies from Ethiopia [26], [28] indicated that prolonged labour showed significant positive association with utilization of institutional delivery services. As the length of labour prolonged the woman want to deliver in health institution. If the first delivery ends safe at home then the probability of going to health institution for the next delivery is less likely.

Distance were significantly associated with institutional delivery (AOR: 3.541, 95% CI: 1.732–7.239). Similar studies in low income countries [21], [22], [30], [31] showed that physical distance is one of the major constraints that prevented community members from accessing and using trained attendants and institutional deliveries. Most people will not travel further than 10 km to basic preventive and curative care.

Respondents awareness on skill of health care professionals (AOR: 2.454, 95% CI: 1.663–6.255) were significantly associated with institutional delivery. In Northwest Ethiopia, awareness about health facilities to get skilled professionals was strong predictor of skilled delivery care use [29].

Transportations on foot (AOR: .258, 95% CI: .122–.549) were significantly associated with decreased institutional delivery. Study from Nepal also indicated that availability of transportation and distance to the health facility are factors determining for uptake of skilled birth attendance for delivery [5]. Walking is the predominant form of transportation in rural Africa as a result of the lack of infrastructure and motorized transport services [32]. Few individuals are having a car; there is poor public transport system, transportations even if available it is not suitable for labouring women, poor infrastructure. The stakeholders should work to access transportations like ambulance service; expanding basic and comprehensive emergency obstetric facilities improve institutional delivery in the area.

Four or more ANC (AOR: 2.914, 95% CI: 1.105–7.682) showed association with institutional delivery. ANC visit is associated with facility based delivery in different regions. Studies from some African countries [15], [22], [23], [24], [31] indicated that women with more ANC were more likely to deliver with the assistance of a skilled attendant. Ethiopian demographic and health survey indicated that 34% of women received ANC care but institutional delivery is 10% [1]. It implies that in Ethiopia there are opportunities to improve institutional delivery including issues related four or more antenatal care. Limitation of the study was case control studies are not able to establish temporal relationships. Some items are used instead of total score which can be the limitations of the study.

## Conclusions and Recommendations

Secondary and above education, duration of labour, living in urban area, ANC care utilization, birth preparedness, reported pregnancy related problems, skill of health care professionals were significantly associated with increased institutional delivery. Therefore, improving women’s educational opportunities is very important, which in turn will enhance the use of delivery care services. This can be achieved as a long-term action but could also be achieved by health education program by addressing more women with no education.

Higher family size, higher birth order, distance from the health institutions, transportations on foot were significantly associated with decreased institutional delivery. Policy makers, health service organizations, community leaders and other concerned bodies have to consider the predictors of institutional delivery to improve skilled attendant in the area.

We recommended that there should be progress toward a health education program that enables more women to utilize delivery care. To meet the goal, this program should target specific groups, including rural and uneducated women, through appropriate media. It should also target mothers with higher birth orders and should encourage more use of antenatal care during pregnancy.
